# We care but we’re not carers: perceptions and experiences of social prescribing in a UK national community organisation

**DOI:** 10.1177/17579139231185004

**Published:** 2023-07-25

**Authors:** B Porter, C Wood, P Belderson, C Manning, R Meadows, K Sanderson, S Hanson

**Affiliations:** School of Health Sciences, University of East Anglia, Norwich Research Park, Norwich, NR4 7TJ, UK; University of Winchester, Winchester, UK; Norwich Medical School, University of East Anglia, Norwich, UK; Sheddington, London, UK; UK Men’s Sheds Association, Bristol, UK; Professor, School of Health Sciences, University of East Anglia, Norwich, UK; School of Health Sciences, University of East Anglia, Norwich, UK

**Keywords:** social prescribing, men’s mental health, loneliness, isolation

## Abstract

**Aims:**

(1) To explore how social prescribing referrals impact experiences of existing members of a voluntary and community-based organisation and (2) to describe the processes and relationships associated with joining community and voluntary organisations.

**Methods:**

Online survey and qualitative interviews with members of Men’s Sheds, a global volunteer-led initiative to address loneliness and social isolation in men. 93 self-selecting Shed members (average age 67 years, 93% male) from across England and Scotland took part in the survey about demographics, joining the Shed, and free-text questions about experiences in the Shed. From the survey participants, 21 Shed members were purposively sampled and interviewed to explore the impact of social prescribing and referrals on the Sheds.

**Results:**

Participating in the Men’s Shed was often associated with a significant change in personal circumstances, and Sheds provided a unique social support space, particularly valuable for men. Key factors around experiences of social prescribing and referral mechanisms were identified. We developed three themes: the experience of joining a Shed, success factors and risks of social prescribing, and ‘we care but we’re not carers’.

**Conclusions:**

The results show that Men’s Sheds are a caring organisation, but their members are not trained as professional carers, and men come to the Shed for their own personal reasons. They are concerned about the potential additional responsibilities associated with formal referrals. They encourage the development of relationships and local-level understanding of the essence of Sheds to enable social prescribing. As models of social prescribing grow nationally and internationally, collaboratively working with voluntary and community organisations to develop a mutually beneficial approach is essential for the effectiveness and sustainability of social prescribing in community health.

Problematic levels of loneliness are experienced worldwide.^1^ Up to 18% of the UK population often feel lonely,^2^ and there are 1.2 million chronically lonely older people.^3^ The UK government had taken action to tackle loneliness prior to the Covid-19 pandemic; appointing a Minister for Loneliness (2018), launching a loneliness strategy (2019) and in England, investing over £4.5 million in social prescribing schemes to tackle loneliness, social isolation, and the associated chronic health implications.^4^ There are several different models of social prescribing in England, such as social prescribing link workers funded by regional health and social care commissioning organisations to work in the regions’ primary care practices, working with primary care patients. Most models have a link worker who works with individuals to access local support.^5^ Social prescribing applies a social model to health, enabling person-centred and holistic care systems, and similar models have also been applied in the US^6^ and Canada.^7^ Social prescribing takes an asset-based approach, identifying and promoting individual and community needs, capabilities, and resources to support physical, social, and mental health,^8^ through referrals from primary care practitioners, other health professionals, or voluntary and community organisations. For example, if someone had high blood pressure, they could be referred to community support for weight, diet, and physical activity through a walking group,^9^ or if someone were socially isolated, they could be referred to a knitting or crochet group for social and craft activity.^10^

Research into social prescribing in England found positivity, commitment, and support^8^ although the experience varied greatly in different areas of the country. The evidence has been criticised as being non-robust and heavily reliant on qualitative reports.^11,12^ Despite the wide variation in approaches to social prescribing, there have been decreases in loneliness, improved mental wellbeing, and increases in social connections and overall wellbeing.^13,14^ Social prescribing has the potential to address a large unmet need for non-medical interventions to support population health and reduce burden on health care.

Voluntary and community organisations are key to enabling social prescribing. One example, with a particular focus on men, is the UK Men’s Sheds Association (www.menssheds.org.uk). Men’s health is an underrepresented area of public health policy, literature, and service provision.^15^ Research has highlighted the importance of gendered approaches, like Men’s Shed, to engage men with health and mental health and the impact of the ‘right’ context that considers differing male behaviours and attitudes.^15,16^ This project was funded as part of the University College London Loneliness and Social Isolation in Mental Health Network to explore interventions to reduce loneliness and social isolation and promote mental health. Men’s Sheds were selected for this project as an example of a community-based intervention to tackle loneliness and isolation in men, a group that is underrepresented in voluntary service provision, at particular risk of poor mental health, and less likely to access psychological support. Social prescribing might show some promise as being an appropriate mechanism for targeted engagement and inclusion of men who are of low mood, in poor mental health, and isolated, but this is not known. Men’s Sheds (Sheds) are ‘making environments’ where men (and women) come together in a social environment to engage in practical projects. Originating in Australia, Men’s Sheds are a global initiative, and there are over 600 Sheds in the UK. Typically, members of Men’s Sheds are retired or unemployed and often older (over 65 years), but the reported age of members has ranged from 18 to 91 years.^17^ Men’s Sheds are commonly peer-led, run by volunteers who are older, retired men who also make use of the facilities themselves. In the UK, most Sheds are self-funded and will need to apply for charitable funding to set up and sustain the Shed, the premises, and upkeep of the Shed. Some Sheds will supplement funds by making and mending things for people and groups in their community for a small fee. Sheds support their members through shared activities, offering new social relationships, and often contributing to community projects. Sheds take a ‘shoulder-to-shoulder’ rather than ‘face-to-face’ approach, recognising that many men feel less comfortable discussing personal matters, such as mental and physical health issues, and are more reluctant to seek support or disclose concerns to friends and family.^18^

Research has explored the impact Sheds have on mental health, physical activity, and loneliness.^17,19,20^ Sheds can impact health behaviours and attitudes of Shed members through an inclusive environment of practical and social activities.^21^ Recently, a health-promotion initiative, ‘Shed for Life’, was trialled in Ireland.^22^ The trial examined if Sheds ought to be utilised as places for health-promotion and social prescribing initiatives. While the Shed members felt comfortable discussing physical health, talking about mental health was more difficult. Sheds are concerned about the potential for stigmatisation as mental health support providers and formalising a responsibility for providing peer support.^23^ The study reported here sought to address these matters further by exploring how people are introduced to Sheds (including processes such as social prescribing), how these processes impact experiences, and how best to promote Sheds as an offer for potential health-promoting interventions.

The aims of this research were (1) to explore how social prescribing referrals impact experiences of existing members of a voluntary and community-based organisation (Men’s Sheds) and (2) to describe the processes and relationships associated with joining community and voluntary organisations. This study received ethical approval from Faculty of Medicine and Health Sciences Research Ethics Committee (2020/21-110).

## Methods

### Survey with Shed members

Shed members from across the UK were invited to take part in an online questionnaire and virtual interviews (April–October 2021). The research was promoted through newsletters and social media via UK Men’s Shed Association (UKMSA), providing an information sheet and expressions of interest. Any Shed member could take part if they were a current member, older than 18 years, and provided consent.

The questionnaire included questions on demographics, Shed characteristics, joining the Shed, and free-text response questions relating to their experiences being part of the Shed. 93 Shed members took part in the questionnaire.

### Interviews with shed members

From the questionnaire sample, a sub-section was purposively sampled and invited to take part in semi-structured interviews to explore perceptions, views, and experience of referrals, including social prescribing, in Sheds (topic guide and survey questions available in online Supplemental Material). We explored both the experiences of joining a Shed through informal ‘referral’ routes (such as through a relative or spouse) and the experience of people being formally referred to the Shed through social prescribing schemes. The purposive approach was to sample an equal split of Shed ambassadors (*n* = 10) (Shed ambassadors are Shed members who additionally volunteer with UKMSA to contribute to local, regional, and national development of Sheds, as advocates for the Shed movement) and Shed members without ambassador roles (*n* = 11). Shed ambassadors are often actively involved in the promotion of Sheds and their perceived benefits, and so we wanted to ensure a wider variety of experiences of Sheds were also included. We also aimed to sample to ensure representation of women (*n* = 3), people living with disabilities (*n* = 6) and mental health conditions (self-identified) (*n* = 5), and a range of Shed sizes and locations (urban *n* = 11 and rural *n* = 10).

Survey and interview questions were first piloted with the UKMSA Health and Wellbeing Committee (six Shed members). Interviewees were informed about the topics prior to the interview, and interviewees had no previous relationship with the researchers. Interviews lasted between 30 and 120 minutes, via videoconferencing (Zoom or Microsoft Teams) and were conducted by two experienced female, white post-doctoral researchers (B.P. and P.B.). Interviews were transcribed verbatim, checked by the researcher, de-identified, and recordings destroyed.

### Analysis

Survey and interview data were analysed using an inductive thematic framework approach to understand experiences and reasons for joining the Shed and perspectives around referrals and social prescribing.^24^ An inductive approach looks for patterns in the interpretation of data that can be used to generate an explanation.^25^ We allowed the data to determine the initial themes, which were then developed into a framework that was used to guide the subsequent analysis of transcripts. Five interviews were independently coded by B.P. into broad themes, and following the initial inductive analysis, a broad coding framework was created to guide independent analysis of the remaining transcripts by C.W. and S.H. There was continual discussion among the research team about the interpretation of the data and refining the themes. An example of how the analysis progressed is provided in the Supplemental Material. Data were managed using NVivo 12 (QSR International Pty Ltd, 2018).

## Results

93 Shed members completed the online survey. The average age was 67 years (range 41–91 years), 93% were male, and 99% white. Seventeen (18%) had a physical disability, and 11 (12%) a mental health challenge (self-identified). Twenty-seven (29%) were Shed ambassadors, and 83% were members for more than two years. The sample was broadly representative of the national picture of Men’s Sheds.

28 survey respondents were invited to be interviewed, and 21 took part. Interview participants represented 21 different Sheds, average age 66 years (range 41–77 years), 18 men, and 20 were white British (reflecting the sample composition). Six people had a disability, five a mental health challenge, and ten were Shed ambassadors. The interviewed sample covered England and Scotland, and broadly half were in rural areas (*n* = 10) and urban areas (*n* = 11).

From our analysis of the free-text responses in the questionnaires and the interview data, we developed three themes: experience of joining a Shed; success factors and risks of social prescribing; and ‘we care but we’re not carers’. These themes are explored further below. The interview quotes are provided with pseudonyms.

### Experience of joining a shed

There were many pathways to, and motivations for, joining a Shed. Joining sometimes involved an informal ‘referral’ or signposting by a relative, spouse, friend, or via another group in the community. There were some instances described in the interviews of referrals via formal social prescribing routes.

“Well, what happens is we get an email saying they have a specific person they think would benefit from our Shed and then we also we get an email or a phone number or something for them. They’ve already talked to them about it, and we just invite them in to come and have a look down and see what they think. And you know it’s amazing how some people, they’ve all got issues, but when they come in – we have a laugh, a joke most of the time anyway – but it’s funny how quickly they feel like they belong.” (Interview participant, Ray, Shed Member, age 59)

Often, joining the Shed was associated with a considerable change in personal circumstances, bereavement, retirement, moving home, and experiencing a medical diagnosis (e.g., dementia) or a physical injury.

“I was dealing with feelings of morbidity and depression following a number of bereavements and was trying to help myself get back to some useful state of health mentally and physically. I tried a number of avenues to help myself including GP, bereavement counselling, mental health counselling, weight loss program. The Shed has been the most beneficial.” (Survey participant)

“I’ve been diagnosed with vascular dementia, and I just needed to do things to keep me as independent as possible.” (Survey participant)

Retirement changed an individual’s day-to-day routine and lifestyle, and the transition could be challenging. The Shed provided a new sense of purpose and value.

“Having left a full-time and busy job due to retirement, I suddenly had no purpose outside of my family life. The switch was too great, and I suffered badly with some sort of depression. I saw a poster for the local Shed and went along.” (Survey participant)

Being part of a Shed helped to create new identities, that were particularly important to people after they had retired, for example:

“I’m a Shed ambassador and I run my own Shed and people go ‘what’s that?’ Shed gives a new identity instead of saying I used to be a policeman or in the Navy.” (Interview participant, George, Shed member and Shed ambassador, age 76 years)

Shed members wanted to meet like-minded people, make friends, reduce isolation, engage in their local community, and ‘have a laugh’. Being part of the Shed helped them to develop new skills, use tools and equipment, continue, or develop their skills in making and mending things, and for some, help others and share their own skills. During the Covid-19 lockdowns and restrictions, Sheds endeavoured to provide a source of contact and support, often through virtual meetings or emails.

“The most amazing thing to discover is how much humour exists in a men shed, all the guys without exception have got a sense of humor and we have a laugh and that’s the that’s the levity that we want to keep.” (Interview participant, Logan, Shed Member, age 70)

### Success factors and risks of social prescribing

The participants we interviewed were aware of social prescribing but had a mixed experience of referrals in their Sheds. ‘Some successes’ (interview participant Murray, Shed member and Shed ambassador, age 77 years) were described where Sheds had several referrals and good links with social prescribing in their area. Other participants were not aware of any referrals to their Shed, but they knew their Shed had been contacted by local social prescribers, and in some cases, there had been no referrals and no contact.

Examples of referrals included ex-service men, young adults, people living with autism, terminal illness, dementia, after stroke, mental health conditions, and informal referrals from relatives (often wives). The Shed enabled opportunities to share skills and knowledge, learn from others, and gain confidence and informal support.

Where it worked well, it was based on having good relationships with prescribing organisations that understood the Shed’s and individuals’ needs and capabilities. This then tended to set a good example for future connections.

“The referrals we get, they’ve been very good from [community development trust]. They’ve not just chucked people our way, it’s the ones that they feel that can get some benefit. So, we don’t get problems or stumbling blocks because the people we get is suited to what we are.” (Interview participant, Ray, Shed member, age 59 years)

Some Sheds had experienced additional benefits of linking with health professionals to enable health-promotion initiatives within their Sheds through a Link Worker.

“I mean one of the Shedders wanted us to make a coffin, his own coffin. And so, one of the link workers was in the Shed and heard we were making the coffin and she said, well, how many of your Shedders had an end–of–life plan? And I said, well, I don’t know – why don’t you bring the end-of-life planning stuff in and talk to the guys that are making the coffin? And so, she ended up coming in and talking to six or seven Shedders about the end of last month.” (Interview participant, Tony, Shed member and Shed ambassador, age 77 years)

There were, however, multiple negative reports of experiences of social prescribing referrals into Sheds that had left the participants viewing social prescribing with caution. Shed members had previous experiences of their Shed feeling like a ‘dumping ground’, and those inappropriate referrals had been ‘thrust upon them with an illness or a disability’ (interview participant Esther, Shed Member, age 77). Often, this included cases where a referral had been made without prior consultation with the Shed, they were not made aware of additional needs, or where new members arrived without someone to assist them in their first few sessions.

“The trouble is the carers came in and to our surprise, they left this chap with us. Then they went outside sat in the car, and for two hours and left. And we didn’t think that was good enough. It put pressure on us as individuals. We’re not trained carers and we felt really uncomfortable, so we had to say we don’t think this is gonna work. It’s too dangerous.” (Interview participant, William, Shed member and Shed ambassador, age 63 years)

“They see us as a means to an end, but they’re not interested in the effect on us.” (Interview participant, Murray, Shed member and Shed ambassador, age 77 years)

Notably, while all participants recognised the value of social prescribing and could appreciate why Sheds may be a suitable place for referrals, they felt the decision to be involved could not be enforced and should be led by each Shed, to enable the Shed’s unique capabilities and capacity to be carefully considered on a case-by-case basis.

“I’d certainly like to have those referrals in place, but it has to be as I said, it has to be done by the person doing that and knowing that the limitations of the Sheds and the network of Sheds. Because a lot of us are in the same position. It’s just trying to manage people’s expectations around; you know what we can do. You know, suggesting the place maybe somewhere to attend and not knowing those limitations and then that person to be disappointed with it.” (Interview participant, Eric, Shed member and Shed ambassador, age 41 years)

All participants highlighted the practical health and safety challenges of facilitating referrals in Men’s Shed environments. The nature of the activities that are undertaken in the space (e.g. building, woodwork, metal work) often will involve potentially hazardous machines, tools, and materials, and concerns were raised about the additional health, safety, and support requirements particularly if referred individuals required additional support or accessibility needs. This had created tension and concern for the Sheds, and some decided to review their approaches to new memberships.

“The problem is if you’ve got somebody who thinks he can use equipment and we’ve got no means of assessing whether or not that’s true or not. That’s where the danger is. It would be difficult for us to, you know, we would be seen as some nasty sort of autocratic organization if we started laying down laws to individual people when they thought that we’re being unreasonable.” (Interview participant, Ethan, Shed member and Shed ambassador, age 67 years)

Some participants described how they were concerned about additional formalised procedures that would be required if they were to accept social prescribing referrals and how this would impact upon the informal nature of the Shed.

“Some social prescribing schemes want you to fill in a ream of paper. They want you to be on a database of organisations who will accept clients who will support them, who will say they’ve been for how many hours this week? We’ve never wanted to go down that route because we are a membership group. We’ve always tried to keep it as informal as possible and that works for us as a Shed.” Interview participant (Maria, Shed member, age 45 years)

The natural ethos and dynamics of each Shed were very important to participants, and some were concerned about how referring people may impact this, some arguing that arriving through a referral route may impact the motivation of the person referred and the enjoyment of existing members.

“I don’t want it to destroy our Shed.” (Interview participant, William, Shed member and Shed ambassador, age 64 years)

“I do think that referrals are not a good idea. I think if people want to come to the Shed, they should come themselves. There’s nothing wrong with social prescribing telling people about this place but not referring them on. Not saying ‘we’ve got somebody here, we’ll send them’ because you’re going to spoil, no, you could possibly spoil Members’ enjoyment . . . it’s going to be too much of a worry. You’re going to take enjoyment away and people are going to be thinking ‘I can’t go there and relax and have a laugh and mess about and help people because that bloke will be there again and it’s worrying me’.” (Interview participant, Esther, Shed member, age 77 years)

### We care but we’re not carers

An overriding message from the interviews was that Shed members are not formal care service providers, and although they do care, they are not carers, but equals. The Shed creates a place for a mutual exchange of skills and support, a reciprocal relationship where individuals gain new skills, access to tools or equipment, and a sense of community, where they can also give back, such as sharing skills, offering informal support to others, or being part of the administration to organise the Shed. The reciprocal nature of these relationships offers a sense of purpose and value that was important.

“We’ll share their skills with those who have fewer skills and there’s a lovely mutuality about the whole thing. And that balance is a really important part of it.” (Interview participant, Logan, Shed member, age 70 years)

There is an ethos of the Shed as open and a place for support, but discreetly. There is an underlying deep care for each other and their needs, being non-judgmental and without making it overt.

“The atmosphere we create that allows people to come and then see that you know we’re quite open and able to talk about things and. I think quite quickly they learn that they’re not going to be judged by us and it’s alright to just be ‘them’. If they want to rant and shout and rave, you know they can do, you know.” (Interview participant, Ray, Shed member, age 59 years)

Sheds and Shed members will offer a place for support, but their very nature defines them as a place where their members are also using a space they view as safe and an ‘escape’ from their usual day-to-day responsibilities. Shed members are not trained to care for people with additional needs (and the impression was that they would not want to be trained), and they did not want to be required to be responsible.

“Men’s Sheds are a caring organization, but we are not carers we do not have the staff expertise or training to be carers. So, anyone with any disability can be a member of the Shed, but only if they are safe on their own. . . The headline issue is safety for the person and for other people around.” (Interview participant, Bernard, Shed member, age 71 years)

## Case Studies

Four case studies were created from the information given from the 21 interviews. These can be found in the online Supplemental Material provided to help the reader contextualise the findings and better understand the experiences of Sheds, referrals, and social prescribing. The personalised details of the case studies have been generalised from the interviews to ensure the anonymity of sheds and individuals.

## Discussion

This study has explored the experience of social prescribing referrals of existing members of Men’s Sheds. This organisation is an example of a volunteer-led community organisation with the potential to be a suitable fit for the increasing use of social prescribing in supporting health, mental health, and wellbeing and easing burden on health and social care services. However, our findings identify key areas of concern arising from members of Men’s Sheds that have broader applicability for other voluntary organisations, including taking ownership of people’s welfare, not being trained professionals, and that a referral system could disrupt the natural ethos and dynamic that is vital to any voluntary organisation. When referrals are made, having established good relationships and understanding of the Shed, the potential and value of social prescribing is recognised. Like many other voluntary organisations, Men’s Sheds are a caring organisation, but they are run by volunteers who come for their own personal reasons. Social prescribing is becoming an integral part of the health and social care infrastructure, and voluntary and community groups are already feeling the effects, not always to their benefit.

This study also found that joining a Shed was often associated with time of considerable change in personal circumstances and that Men’s Sheds offered a unique setting for men to access support and to be comforted by the unique group dynamics. Understanding how groups facilitate change is important for the development and design of interventions.^26^ Our findings highlighted the natural essence that develops in Sheds and that there are many characteristics that contribute to positive outcomes for members, including (but not limited to) group composition, dynamics, purpose, and social and personal factors and setting.^27^ Social prescribing and the Shed movement have an asset-based way of thinking about health promotion and inequalities. Instead of a deficit approach to ill health, it is one of ‘salutogenesis’ (focusing on what makes people well rather than a deficit model of ‘what is wrong’ with people’s health).^28^ Taking a salutogenic approach to designing and commissioning interventions may help to enable the desired outcomes.^29^

Both social prescribing and Men’s Sheds are aimed at reducing loneliness and isolation. Sheds may be an ideal place for referral for some men.^21^ Sheds are a suitable place for health-promotion initiatives, as indicated by the ‘Shed for Life’ project in Ireland that brought a tailored health-promotion programme into Men’s Sheds and found positive physical and mental wellbeing outcomes and increases in social relationships. The ‘Shed for Life’ programme was co-designed, building important trusting relationships and enabling the tailoring of priorities for the members.^30^ Moreover, ‘Shed for Life’ highlighted the potential that co-designed men’s health initiatives could have in transforming stereotypical gender norms, normalising reflective engagement with health and wellbeing, in an informal space.^31^ Future research can learn from the ‘Shed for Life’ model to explore opportunities for co-designed health-promotion interventions for men in community organisations.

Social prescribing relies on the assumption that voluntary and community organisations can be incorporated into formal care provision.^32^ As we found in Men’s Sheds, this leaves both the organisations and prescribers with a sense of uncertainty and at risk of inappropriate referrals from mental health or social services to ill-equipped voluntary organisations.^33,34^ Since Covid-19, there has been an increase in referrals of 62% to an already under-resourced voluntary sector. There are many factors that aid the enrolment, engagement, and adherence to social prescribing referrals.^35^ Voluntary and community organisations and health professionals both argue that for social prescribing to be effective, funding and support is needed to develop a sustainable model.^36,37^ The introduction of integrated care systems (ICS) (partnerships of health and care organisations to plan and deliver health services) in England could help to build stronger partnerships with voluntary and community organisations. An ICS could provide the infrastructure to develop sustainable approaches to social prescribing by working with local voluntary organisations, primary care, referring organisations and local commissioners to develop approaches for sustainable social prescribing, and incorporating the voluntary and community sector perspectives.^38^

### Strengths and limitations

Men’s Sheds are increasing in their presence across the UK, are uniquely placed to target the inclusion of men, and offer a good representation of the experience of community-based, voluntary organisations who are increasingly at the interface with social prescribing. Although this study provides an important perspective on the experience of a community organisation, there are limitations, including that we could not recruit new Shed members (less than 6 months), Shed members who had been referred through social prescribing schemes for interview. Future research should explore the perspectives of people who are referred to Sheds through social prescribing, particularly among those who do not fit the usual demographics of Shed members (older than 65 years and retired).^16^

## Conclusion

Men’s Sheds are a community organisation that functions through the commitment of volunteers and charitable funding. With the growth of social prescribing, the voluntary and community sector is increasingly feeling pressure from a lack of infrastructure support, funding, and resources to support sustainable organisational development that can effectively support social prescribing referrals. Without support, funding, and trusted local-level referrer-provider relationships, community organisations like Men’s Sheds may find it difficult to engage. The sustainability and effectiveness of social prescribing rely upon the availability and quality of the organisations that individuals can be referred to, and it is imperative that their perspectives are accounted for when developing approaches to asset-based community support in the future. To retain the special and discrete roles that organisations such as Men’s Sheds have in our communities, social prescribing initiatives must proceed with caution so they do not destroy the very essence of the thing they wish to use as a panacea for the increasing number of people who may benefit from non-medical interventions.

## Supplemental Material

sj-docx-1-rsh-10.1177_17579139231185004 – Supplemental material for We care but we’re not carers: perceptions and experiences of social prescribing in a UK national community organisation Supplemental material, sj-docx-1-rsh-10.1177_17579139231185004 for We care but we’re not carers: perceptions and experiences of social prescribing in a UK national community organisation by B Porter, C Wood, P Belderson, C Manning, R Meadows, K Sanderson and S Hanson in Perspectives in Public Health

sj-docx-2-rsh-10.1177_17579139231185004 – Supplemental material for We care but we’re not carers: perceptions and experiences of social prescribing in a UK national community organisation Supplemental material, sj-docx-2-rsh-10.1177_17579139231185004 for We care but we’re not carers: perceptions and experiences of social prescribing in a UK national community organisation by B Porter, C Wood, P Belderson, C Manning, R Meadows, K Sanderson and S Hanson in Perspectives in Public Health

sj-docx-3-rsh-10.1177_17579139231185004 – Supplemental material for We care but we’re not carers: perceptions and experiences of social prescribing in a UK national community organisation Supplemental material, sj-docx-3-rsh-10.1177_17579139231185004 for We care but we’re not carers: perceptions and experiences of social prescribing in a UK national community organisation by B Porter, C Wood, P Belderson, C Manning, R Meadows, K Sanderson and S Hanson in Perspectives in Public Health
