# Supplementary material for: We care but we’re not carers: perceptions and experiences of social prescribing in a UK national community organisation
Source: Perspect Public Health. 2023 Jul 25;145(3):167–74. doi: 10.1177/17579139231185004 (PMC12231811; doi:10.1177/17579139231185004)
Supplement: sj-docx-1-rsh-10.1177_17579139231185004 – Supplemental material for We care but we’re not carers: perceptions and experiences of social prescribing in a UK national community organisation [file sj-docx-1-rsh-10.1177_17579139231185004.docx]

**Supplementary Material**

**We care but we're not carers: Social prescribing case studies in Men's Sheds**

***Names removed until publication***

**2023**

The four case studies are designed to complement thematic analysis by illustrating typical scenarios derived from the interview data. The case study vignettes were created to illustrate pertinent issues, and are anonymous and composite, use fictitious names and are based on a blend of stories derived from interviews which shared similar features.

‘Jamie’

Jamie, a young man with autism, who was not in education or work, arrived at his local Men’s Shed after a referral via his GP who had suggested it to his parents as something which might benefit him. This Shed had good links with the social prescriber at local GP practice (“w*e are known about in the local surgeries […] It's on their radar”),* who was also a member of the Shed committee, and as such had good understanding of the Shed itself.

Jamie attended his first sessions at the Shed alongside his father.  He was warmly welcomed by a Shed member who made an effort to spend time chatting with Jamie and his Dad, talking through the alternative activities he could try when he felt ready, and asking him what he might be interested in.

At first the Shed environment was quite overwhelming for Jamie: *“He kind of almost hid in the corner. And if it got too noisy he had to go.”* However, over time, and with the gentle encouragement of other Shed members, Jamie began to feel better able to participate: “*The lads were really good with him, they talked to him and he started to come out of himself a little bit.”* With the support of the Shedders, Jamie adapted and began to find a niche in woodwork: “*He became quite adept at the lathe which surprised him and us, and he did very well and his confidence increased enormously.”*Over time, his father no longer needed to accompany him and he began arriving independently at the Shed every week.

Jamie eventually stopped attending the Shed because he found employment.  Though sad to see him go, members were delighted to have played such an integral role in his journey towards independence: “*If it hadn't have been for the building confidence that he got through being in the Shed he wouldn't have got a job, so that was a good outcome. It's a good way to lose people.”*

**Learning points:**

A number of factors helped make this referral work, including:

- Good local-level working relationship with prescriber/referring body, including an understanding of the nature of and capacity of the individual Shed to support the person who is referred
- Unique and transformative setting that Men’s Sheds can provide
- Inclusion of a transition period whereby accompanying carer was in attendance while a new member settled in
- Welcoming and sensitive support from existing Shed members
- A range of activities available at the Shed and gentle pace of integration – ‘making it work’ for the individual.

‘Henry’

Henry was referred to the Shed via his GP surgery, following a head injury, which had affected his cognitive functioning.  The Shedders had little information on the context of the referral beforehand and were unprepared for the level of support needs encountered.

A few weeks in, Henry began displaying volatile and abusive behaviour, which the Shedders felt unable to handle, including one upsetting episode where “*He turned violent, really violent. A lot of people were worried. Where he was standing, there's a lot of hammers and stuff behind where he was standing shouting at people. People were worried he was gonna start slinging stuff around the Shed.”*

As a result of this, some members stopped attending, fearful for their own safety, and concerned about the implications of having to manage the disruptive behaviour.  The Shed temporarily closed while the matter was considered and Henry was asked to terminate his membership: “*We couldn't have any conflict there 'cause our members are vulnerable.”*

This was not an easy decision for the Shed to make, balancing their wish to include and support new members who need additional support against their own capacity to be able to offer the level of help required.  This was also juxtaposed alongside the risk of undermining the established healthy dynamic of the Shed: “*It's a really horrible dilemma…We're not proud of it, but it was a for the benefit of the majority [...] There's a lovely mutuality about the [Shed] and we don't want to be doing mental health nursing.”*

The situation prompted the Shed to review their entire membership process, incorporating an application that includes signing up to core values (e.g., no abusive behaviour, or attending under the influence), and exploring what other services were available to signpost to for members who they felt unable to support. A probationary period was also introduced: “*They come as a guest for a few weeks where they are assigned someone to look after them and help them and guide them and at the end of that period there will be a discussion about whether they should progress to full membership. It needs to be right for them as well.”*

Following this experience, the Shed have become wary of social prescription.  However, they were prepared to consider engaging with referrals in the future if they felt assured that social prescribers could put an effective screening process in place, combined with a full understanding of the nature of and capacity of the Shed itself:   *“If [link worker] would visit the Shed, they would need to participate to understand the ethos and the flavour of the Shed; and they would have the big responsibility of screening potential members, we might be prepared to help, but only after discussion […] all of us have a social conscience, we would not want to not help somebody. We do.  But there are going to be extreme cases which we are not qualified to help with and it's only the link workers who can do that screening process.”*

**Learning points:**

- Shedders are not formal care providers
- Careful and difficult balance to be made between supporting new members and maintaining healthy dynamic of Shed for existing members
- The Shed was compelled to learn and evolve in a reactive way – in response to situation which impacted very negatively on members
- The need for established joined-up working relationships with social prescribers to mitigate against problems, and to ensure the match works both for the Shed and person referred.

**‘Richard’**

Richard, a 32-year-old veteran, was first referred to the Shed through his rehab facility. *“He was in the bomb squad, and he was in a wheelchair, all of his team had died in Afghanistan.”* When Richard first joined the Shed, one or two members of the Shed were briefed about his background by a member of staff from the referring rehab facility. Richard “*suffered severely at times with PTSD and [was] welcomed with open arms.”*

Richard *“was very quiet”* when he first joined but he started working on small projects and was quickly recognised as being very useful and showed a lot of potential to the other Shed members. Richard stated to feel that he was “*doing useful things as well as contributing to the Shed”.*

The other Shed members were kind to Richard and included him in their light-hearted jokes and workshop banter. Quite soon, it looked like Richard could be a good supervisor in the Shed and was enrolled on other courses to train him to do so.

At this Shed, it was quite common that their members were *“largely retired men... and ex-service people... people who’ve left the military and [were] finding the transition into civilian life somewhat difficult”.* The Shed had a good *“understanding and connection”* with their local referring organisation and they knew that *“if at any time they had referred someone who wasn’t right, we’d be able to ring them and say so”.*

For the Shed members, it was clear that Richards confidence had grown, *“just doing one thing leads on to another, leads on to another achievement and [his] confidence is growing and it’s really nice that we’re part of their journey as they’re progressing.”*

**Learning points**

- Sheds are welcoming places for support and rehabilitation (including for veterans) and could enable wider support system through relationships developed in the Shed, as desired.
- The natural essence of Sheds that can enable light-hearted ‘banter’ and camaraderie among members.
- The unique and transformative setting that Men’s Sheds can provide.
- Welcoming and sensitive support from existing Shed members.
- A good working relationship with referring organisations, who understand the nature of the Shed and includes the Shed feeling that they can say ‘no’.

**‘George’**

The Shed received a call from a social prescriber about referring ‘George’, a man in his mid fifies, with dementia, to the Shed. The Shed were initially very cautious about accepting the referral.

The Shed had previous experiences of people with dementia in the Shed, this had included one instance when a wife of someone with dementia had been encouraged to get their husband to try the Shed so they could have some rest bite. However, the wife dropped their husband at the front door and said *“I’ll pick you up in a couple hours, I’ll do some shopping... She went back and her husband was nowhere to be seen,”* as the husband had walked away from the Shed and was lost. The Shed members were understandably very concerned about this incident and about the responsibility placed on them: *“Yes, we agreed that we're we are a caring organization, but we're not carers.”*

After this, the Shed adopted a policy that someone with additional needs must be accompanied by another person (friend, relative, carer, for example). However, this did impact the number of people who could fit into the small workshop space and sometimes meant others missed out.

In the past, the Shed members had felt like their Shed had been viewed as a *“social club, sit down with coffee and natter”* type of place. Previously they’d had calls with social prescribers where they’d explained that a person attending needs to be able to *“be aware of his surroundings and to be able to keep himself out of danger.”* Things were working better for the Shed now as it was felt that referring groups had a better understanding of *“what Sheds are about”* and could more appropriately refer people in.

When it came to ‘George’, the social prescribers knew him well and could comfortably say that he was in the early stages of dementia and was looking for a place for company now that he was no longer able to work. George would be accompanied by a support worker to the Shed, and it was suggested that George and the support worker come at a quiet time, in the first instance. This meant that George could see what was on offer at the Shed and get to be familiar with the space, without lots of noise.

George and the support worker both became members of the Shed and attended regularly. George could help with some woodwork projects and often told jokes to the group. He really enjoyed having a space to have a laugh with other blokes.

**Learning Points**

- There are important health and safety considerations when referring to Sheds and the safety of all members is paramount.
- Shed policies may be adapted to suit the referral process each individual Shed may choose to take. This is an individual Shed decision.
- Shed members care deeply but they are not formal care providers.
- Social prescribers with a good relationship with the Shed and a good understanding of what the Shed can offer, and the needs and abilities of the person they are referring enables more effective referrals.
