# Supplementary material for: We care but we’re not carers: perceptions and experiences of social prescribing in a UK national community organisation
Source: Perspect Public Health. 2023 Jul 25;145(3):167–74. doi: 10.1177/17579139231185004 (PMC12231811; doi:10.1177/17579139231185004)
Supplement: sj-docx-2-rsh-10.1177_17579139231185004 – Supplemental material for We care but we’re not carers: perceptions and experiences of social prescribing in a UK national community organisation [file sj-docx-2-rsh-10.1177_17579139231185004.docx]

**Supplementary Material**

Example of Analysis Progress

| **Code** | **Quote** | **Theme** | **Theme description** |
| --- | --- | --- | --- |
| Joining the Shed | *Well, what happens is we get an email saying they have a specific person they think would benefit from our Shed and then we also we get an email or a phone number or something for them. They’ve already talked to them about it and we just invite them in to come and have a look down and see what they think.* ***Interview participant (Ray)*** | Pathways and Motivations to Joining the Shed | There are varied pathways to joining the Shed, and different motivations for joining. E.g., sometimes this was encouraged by relatives, sometimes it was associated with a big change in personal circumstances like retirement or bereavement. The Shed can provide a new sense of belonging and identify after the change (e.g., retirement). |
| Sense of belonging | *And you know it's amazing how some people, they've all got issues, but when they come in - we have a laugh, a joke most of the time anyway - but it's funny how quickly they feel like they belong.* ***Interview participant (Ray)*** |  |  |
| Change in circumstances | *I've been diagnosed with vascular dementia and I just needed to do things to keep me as independent as possible* |  |  |
| Sense of identify | *I’m a Shed ambassador and I run my own Shed and people go “what's that?” Shed gives a new identity instead of saying I used to be a policeman or in the Navy.”* ***Interview participant (George)*** |  |  |
