# Supplementary material for: We care but we’re not carers: perceptions and experiences of social prescribing in a UK national community organisation
Source: Perspect Public Health. 2023 Jul 25;145(3):167–74. doi: 10.1177/17579139231185004 (PMC12231811; doi:10.1177/17579139231185004)
Supplement: sj-docx-3-rsh-10.1177_17579139231185004 – Supplemental material for We care but we’re not carers: perceptions and experiences of social prescribing in a UK national community organisation [file sj-docx-3-rsh-10.1177_17579139231185004.docx]

Supplementary Material: Survey Questions

| Thank you for agreeing to be part of this research study. You are free to withdraw or not answer any questions. Withdrawing from the study will not affect your involvement with Men’s Shed or UKMSA. If you have any questions about the research, please contact Bryony (bryony.porter@uea.ac.uk). The survey should take you 10-15 minutes to complete. | | |
| --- | --- | --- |
| Participant and shed demographics.  These are questions about you. Please select or write your answer. | | |
| 1. What is your gender? | | Male  Female  Non-binary  Tans woman  Trans man  Prefer not to say  Other (please state) |
| 1. What is your age? | |  |
| 1. What is your ethnicity? | | White-English / Welsh / Scottish / northern Irish / British / Irish / gypsy or Irish traveller / any other white background    Mixed / multiple ethnic groups - white and black Caribbean / white and black African / white and Asian / any other mixed / multiple ethnic background    Asian / Asian British- Indian / Pakistani / Bangladeshi / Chinese / any other Asian background    Black / African / Caribbean / black British- African / Caribbean any other black / African / Caribbean background    Other ethnic group – Arab / any other ethnic group    Other _ please specify    Prefer not to say |
| 1. Do you identify as having a disability? | | No / yes _ please explain / prefer not to say |
| 1. Do you identify as having a mental health challenge? | | No / yes _ please explain / prefer not to say |
| 1. These questions are about the shed that you’re part of and how you use the shed, please write or select your answer. | | |
| 1. What is the name of your shed? | |  |
| 1. Where is your shed? Please include the postcode, if you know it. | |  |
| 1. How many shedders are part of your shed? | |  |
| 1. Is your shed ‘gender blind’ (do you accept people of all genders)? | | Yes / no / other (please specify) |
| 1. Are you a Shed Ambassador? | | Yes / No |
| 1. How did you first find out about and get involved with the shed? | | Family, friend or relative  My gp, doctor or other health, mental health or care professional referred me to the shed  From another shedder  I found out about the shed myself  Other, please specify |
| 1. How long have you been part of the shed? | | Less than 1 month  2-6 months  6-12 months  1 year – 2 years  More than 2 years |
| 1. We know covid-19 has impacted how you can engage with sheds. How often do you engage with the shed, in non-covid-19 times (e.g. early 2020 / 2019)? | | Less than once a month  1-2 times each month  3-4 times each month  More than 4 times each month |
| 1. Since covid-19 we know that shedders have found new ways to keep in contact with one another, when the sheds have been closed and/or lockdown and social distancing measures have been in place. Has your shed used any of the following methods to keep shedders in contact during these restrictions? | | Telephone calls  Whatsapp or other instant messaging chat groups  Facebook group  Virtual meetings e.g., through zoom or Microsoft teams  None of the above  Other: please specify |
| 1. How often are you able to engage with the shed now, including through virtual platforms, phone calls and conversations with other shedders? | | Less than once a month  1-2 times each month  3-4 times each month  More than 4 times each month |
| 17. Can you recall what brought you to Sheds in the first place? Please explain your answer. | | |
| 18. Overall, how would say being part of a Shed has affected you? | | |

Supplementary Material: Interview Topic Guide

**Let’s talk now about your involvement in the shed –** can you tell me about how you were first introduced to the Shed?

- Where signposted by others – probe:
- Informal / formal referral (e.g. was it a friend or relative or was it from a GP)
- If formal – how did this happen, did you get a letter? Did you have contact with a NHS Link Worker?

What were your first thoughts on finding out about the Shed?

- What was it like for you when you first went to the Shed?

**Social prescribing**

Like you (if formal process e.g. social prescribing scheme) / some people, find out about Sheds through their GP or community health care provider, through something called social prescribing or community referrals.

*Reminder of social prescribing information provided before interview (if needed):*

*Social Prescribing (sometimes known as community referral) is a way for GPs and NHS community healthcare providers to refer or signpost people into local, non-clinical services. This may happen through a contact with a NHS Link Worker or people might self-refer.*

***Social prescribing aims to connect people with existing community-based services or organisations that may help a person’s loneliness or illness and support their wellbeing.*** *It is designed to help reduce the dependency on use of medications and/or reduce emergency admissions to hospitals.*

*Some Sheds already receive social prescribing requests and you may have had contact from a NHS Link Workers in your area.*

Do you know if your Shed is already involved in social prescribing?

Have you had contact from an NHS Link Worker in your area?

How have you found out about social prescribing in your area?

How has / how do you think, social prescribing might impact your Shed?

What positives are there / might there be?

What negatives are there / might you see?

What recommendations might you have for a Link Worker?

That is all the questions from me, thank you for your time so far. Do you have anything more that you would like to add?

Thank you for your time today.

*End recording.*
